# A microfluidic approach towards hybridoma generation for cancer immunotherapy

**DOI:** 10.18632/oncotarget.5550

**Published:** 2015-10-07

**Authors:** Yen-Ta Lu, Gaurav Prashant Pendharkar, Chung-Huan Lu, Chia-Ming Chang, Cheng-Hsien Liu

**Affiliations:** ^1^ Division of Chest Medicine, Department of Internal Medicine, Mackay Memorial Hospital, Taipei, Taiwan, R.O.C; ^2^ Department of Medicine, Mackay Medical College, New Taipei City, Taiwan, R.O.C; ^3^ Department of Power Mechanical Engineering, National Tsing Hua University, Hsinchu, Taiwan, R.O.C; ^4^ Department of Medical Research, Mackay Memorial Hospital, Taipei, Taiwan

**Keywords:** immunotherapy, cell electrofusion, hydrodynamic trapping, dielectrophoresis, LabChip

## Abstract

Dendritic cells/tumor fusions have shown to elicit anti-cancer immunity in different cancer types. However, the application of these vaccines for human cancer immunotherapy are limited by the instable quality and insufficient quanity of fusion cells. We present a cell electrofusion chip fabricated using soft lithography technique, which combines the rapid and precise cell pairing microstructures and the high yield electrofusion micro-electrodes to improve the cell fusion. The design uses hydrodynamic trapping in combination with positive dielectrophoretic force (pDEP) to achieve cell fusion. The chip consists of total 960 pairs of trapping channels, which are capable of pairing and fusing both homogeneous and heterogeneous types of cells. The fused cells can be easily taken out of the chip that makes this device a distinguishable from other designs. We observe pairing efficiency of 68% with fusion efficiency of 64%.

## INTRODUCTION

It is widely believed that tumor antigens expressed in tumor cells can be recognized by immune system and anti-cancer response. Thus, tumor antigens are potential oncotargets for cancer therapy [1]. However, cancer cells may also have numerous mechanisms to evade immune surveillance, such as down regulation of MHC molecules or the antigen processing and presentation machinery, increasing the secretion of inhibitory cytokines, and expressing inhibitory molecules. Thus, there have been ample immunotherapeutic strategies designed to overcome immune tolerance to tumor antigen [2]. Vaccination with dendritic cells-tumor fusions that express broad array of tumor antigens on dendritic cells to induce T cell activation is one of the novel cancer immunotherapeutic strategies [3–5]. Recent advances in use of dendritic cells/tumor fusions to treat cancer patients also have shown some promising results in phase I/II studies. However, the method to effectively accomplish the fusion of differentiated dendritic cells and tumor cells is the key challenge, thus limiting the development of dendritic cells/tumor fusion vaccine [6, 7].

Cell fusion is a powerful tool for biomedical research, including hybridoma generation [8, 9], reprogramming of somatic cells [10], cancer immunotherapy [11] and mammal cloning [12, 13]. Cell fusion is rapidly growing as a promising tool for cancer immunology [14–20] due to its easy implementation, high efficiency, and the high viability of post-fusion cells. Cell fusion can also be achieved in an asexual way by biological media (virus) [21], chemicals (polyethylene glycol; PEG) [22] and physical electric signal (electroporation) [23–27]. However, these methods have been associated with limitations such as toxicity to cells, batch-to-batch variability, and low efficiency.

Electrofusion is a highly efficient, reproducible and non-toxic technique for the application of cell fusion [28]. The electrofusion has advantage of higher cell viability of about 84% compared with tradition PEG based methods which has value of around 80% [29, 30].

However, conventional electrofusion system (e.g. BTX ECM 2001) is bulky and not portable. Also, the use of conventional electrofusion systems has lot of limitations because of its high operating voltage, high probability of multiple cell fusion because of uniform electric field produced between the electrodes [19].

In recent years, cell fusion using microfluidic devices has attracted a lot of attention because of numerous advantages such as precise cell pairing, higher fusion efficiencies, higher cell viability, lower sample contamination and smaller Joule heating effects. Several microfluidic designs, which include enhancement of electric field by protruding electrodes [25, 31–39], field enhancement by microstructure between electrodes, which helps to modify spatial distribution between electrodes [40–44] have been designed.

Regardless of method for cell fusion, higher fusion efficiency depends on perfect cell-cell contact instead of random cell pairing, as is the case in conventional methods. Controlled pairing of partner cell have been demonstrated using a high throughput cell pairing and fusion using chemical conjugation [45], field free micro structure assisted [8, 31], electric field assisted cell pairing with better adoptability using hydrodynamic traps or constriction trapping [32, 33, 36, 37]. Despite availability of various cell fusion platforms, cell fusion chip designing needs a deeper inspection towards performance parameters. Geometry and material of microelectrode are important for efficient fusion process. For example, thicker electrodes help to reduce working voltage and nullifies considerably the drawbacks in traditional electrofusion system, like joule heating, bubble generation, extreme pH condition, and swelling of cells. Correct choice of electrode material helps to have better conductivity, ease of fabrication, biocompatibility and corrosion resistivity. Choice of electronic signal for cell pairing (usually AC signal) or cell fusion (usually DC pulse) is also important. Usually, pulse intensity, duration and pulse number affect the electrofusion efficiency and viability of formed fusion cells. Apart from above mentioned design parameters, cell types and osmolarity of buffer solution are significantly influential factors, which can be considerable impact on cell fusion process [46].

We present here a microfluidic chip, which can rapidly fuse homogeneous or heterogeneous type of cells. The microfluidic chip is easy to fabricate because of its simple design. The traps can be easily arrayed over larger area to get higher number of fused cells. The chip is easy to modify for different size of cells by changing the parameters as discussed in further section of cell pairing channel design. We study here effects of various parameters like effect of flow rate and electric field on trapping efficiency and pairing efficiency respectively. We studied effect of electric field on fusion efficiency to get the optimum values. We studied flow cytometer results for fusion cells as part of post fusion analysis.

## DEVICE DESIGN AND SIMULATION

### Microfluidic chip design

**Figure 1:**
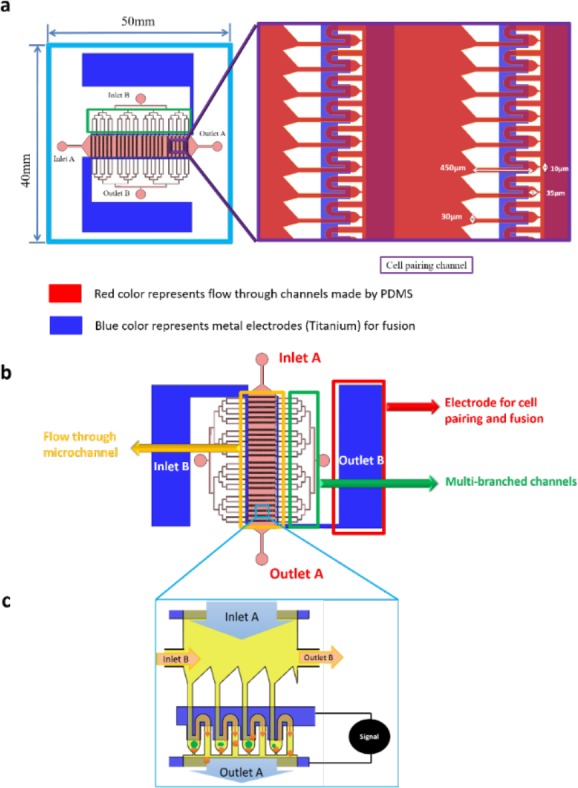
Rapid cell electrofusion device **a.** schematic of microfluidic chip with inlets and outlets and a closer look of cell pairing channel **b.** part wise description of various chambers with magnified view of flow through PDMS channels. **c.** The cells are inserted through Inlet A and taken out from Outlet A. Hydrodynamic resistance created by channel design consisting of a trap with opening of 10 μm helps to catch the cell (shown by orange colour). The second type of cells (shown by green colour) are captured by applying electrical signal. The excess cells are removed by flowing a buffer solution from Inlet B to Outlet B.

Our microfluidic chip is made by a process called soft lithography which consists of use of Polydimethyl siloxane (PDMS). The detailed fabrication process is discussed in latter section. The microfluidic chip consists of total 960 pairs of trapping sites. The cell trapping structure is a flow resistance based trap design with an opening of 10 μm in such a way that it is able to capture a single cell. The designing of microchannels was done after careful analysis of parameters like dielectrophoretic force, analysis of flow resistance, membrane voltage. The device consists of two inlets and outlets each, multi-branched channel and electrode for cell-contact and fusion (Fig. 1b). The cells are loaded from Inlet A and taken out from Outlet A. The Inlet B and Outlet B are used to remove untrapped cells or excess cells, which are present in the channels. The detailed functioning of the chip is discussed in further section.

### Design of cell pairing channels

**Figure 2:**
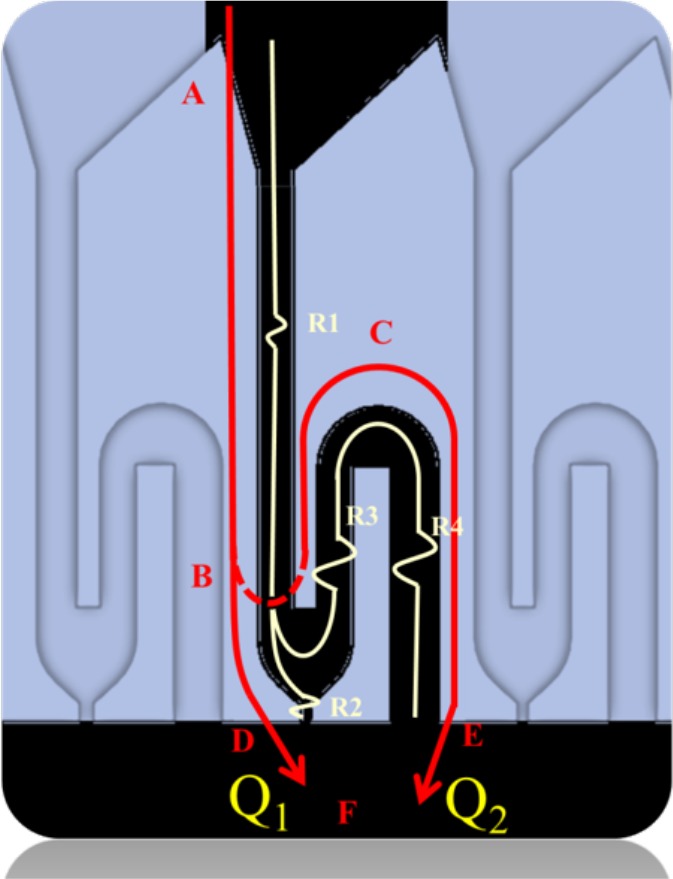
Microchannel composed of by-pass channels and trapping channels with a hydrodynamic trapping site along the trapping channel The pressure drop is defined as Δp, the length of channels as L and the volumetric flow rate as Q.

Presence of cells in electric field for longer duration can damage the cells over period. Thus, to avoid cell damage, we have come up with cell pairing channels which are designed by utilizing dynamic flow resistance and are able to achieve over 960 pairs unit on the chip. The microchannels of device are composed of by-pass channels and trapping channels with a hydrodynamic trapping site along the trapping channel (Fig. 2). Point A to point F included two paths, $\bar{\mathrm{BDF}}$ and $\bar{\mathrm{BCEF}}$. The flow resistances along the channels are R_2_, R_3_ and R_4_. R_1_ is neglected because it is included in both paths. The flow pressure drops along the paths included ΔP_BF_, ΔP_BC_ and ΔP_CF_. Fluid can flow from point B to point F via the channel $\bar{\mathrm{BDF}}$ or via the bypass channel $\bar{\mathrm{BCEF}}$ and the Darcy-Weisbach equation is applied separately for each channel. Because the pressure drops from A to F by both channels are the same, the identity of ΔP_BF_, ΔP_BC_ and ΔP_CF_ can be determined as equation 1

$$\Delta P_{\mathrm{BF}}=\Delta P_{\mathrm{BC}}+\Delta P_{\mathrm{CF}}$$

The flow ratio of the trapping site can be presented as the path Q1 and the by-pass channel Q2. Substitution of equation 1 into Darcy-Weisbach equation leads to equation 2

$$Q_{1}\frac{C(\alpha_{\mathrm{BF}})\mu L_{\mathrm{BF}}R_{\mathrm{BF}}^{2}}{32A_{\mathrm{BF}}^{2}}=Q_{2}[\frac{C(\alpha_{\mathrm{BC}})\mu L_{\mathrm{BC}}R_{\mathrm{BC}}^{2}}{32A_{\mathrm{BC}}^{2}}+\frac{C(\alpha_{\mathrm{CF}})\mu L_{\mathrm{CF}}R_{\mathrm{CF}}^{2}}{32A_{\mathrm{CF}}^{2}}]$$

It is possible to generalize Equation 2 by writing it in the form of a flow ratio of Q1 and Q2 presented as Equation 3.

$$\frac{Q_{2}}{Q_{1}}=\frac{C\left(\alpha_{\mathrm{BF}}\right)L_{\mathrm{BF}}{\left(W_{\mathrm{BF}}+H\right)}^{2}}{C\left(\alpha_{\mathrm{BC}}\right)L_{\mathrm{BC}}{\left(W_{\mathrm{BC}}+H\right)}^{2}{\left(\frac{W_{\mathrm{BF}}}{W_{\mathrm{BC}}}\right)}^{3}+C\left(\alpha_{\mathrm{CF}}\right)L_{\mathrm{CF}}{\left(W_{\mathrm{CF}}+H\right)}^{2}{\left(\frac{W_{\mathrm{BF}}}{W_{\mathrm{CF}}}\right)}^{3}}$$

Here, H is the height of the channels and W is the width of the channels. Cells can be successfully trapped by adjusting the parameters L, W and H and the stream volumetric flow rate and making Q_1_ > Q_2_

**Figure 3:**
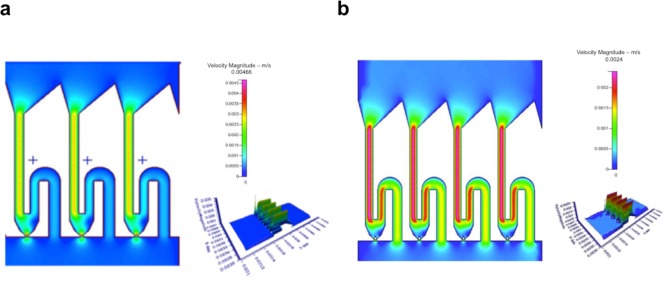
Flow field simulation using CFDRC **a.** fluid velocity without cells trapped in trapping channel **b.** decrease in velocity because of trapped cells in channel.

In order to achieve cell trapping, the width of channel in R_2_ was smaller than the diameter of cell, and the ratio of Q_1_/Q_2_ was determined by adjusting the width of channel in R_4_ since it would not affect the parameters of other channels. In this chip, the Q_1_/Q_2_ was designed as 1.7354 which is critical value for this design. Decrease in value of this would not capture the cells however higher value can trap multiple cells. We verify the cell pairing design parameters by performing simulation using commercial software CFDRC (Fig. 3a, 3b). It is observed that in presence of cells, there is a decrease in the velocity.

### Design of tooth shaped electrode

**Figure 4:**
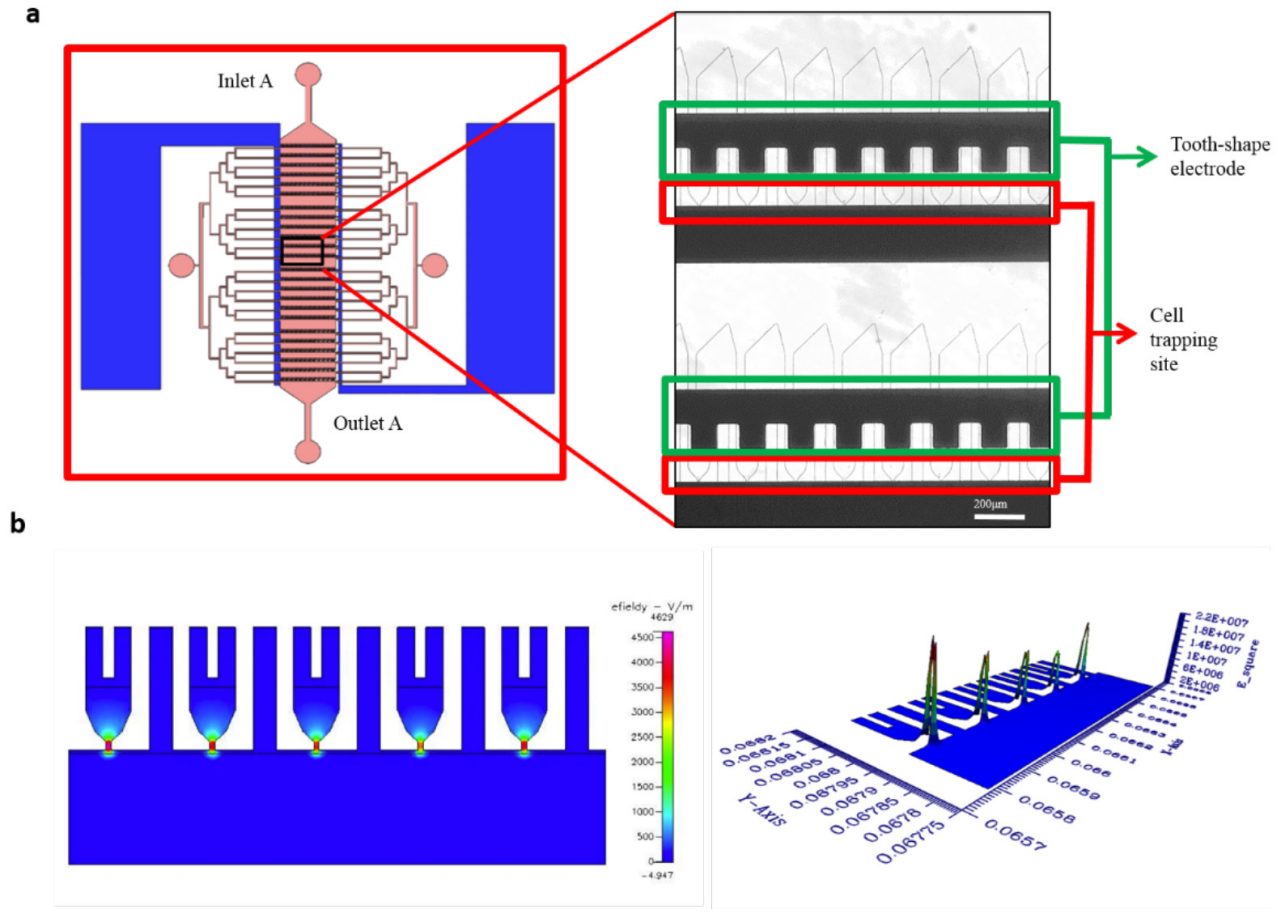
Simulation of electric field generated using tooth shaped electrodes using CFDRC **a.** microscopic image of tooth shaped electrodes **b.** simulation of electric field generated.

The electric field is necessary in cell electrofusion to induce cell perforation and achieve cell fusion. The array with half-tooth-shaped electrodes was designed under the micro-channel to provide electric field for cell pairing and cell fusion. The electrode attracted cells because the structures led the gaps to have relatively high gradient of electric field. The simulation of electric field is performed using CFDRC (Fig. 4a, 4b). The strength of the electric field can be adjusted to control the DEP force and achieve cell electrofusion by inducing cell perforation

## MATERIALS AND METHODS

### Device fabrication

**Figure 5:**
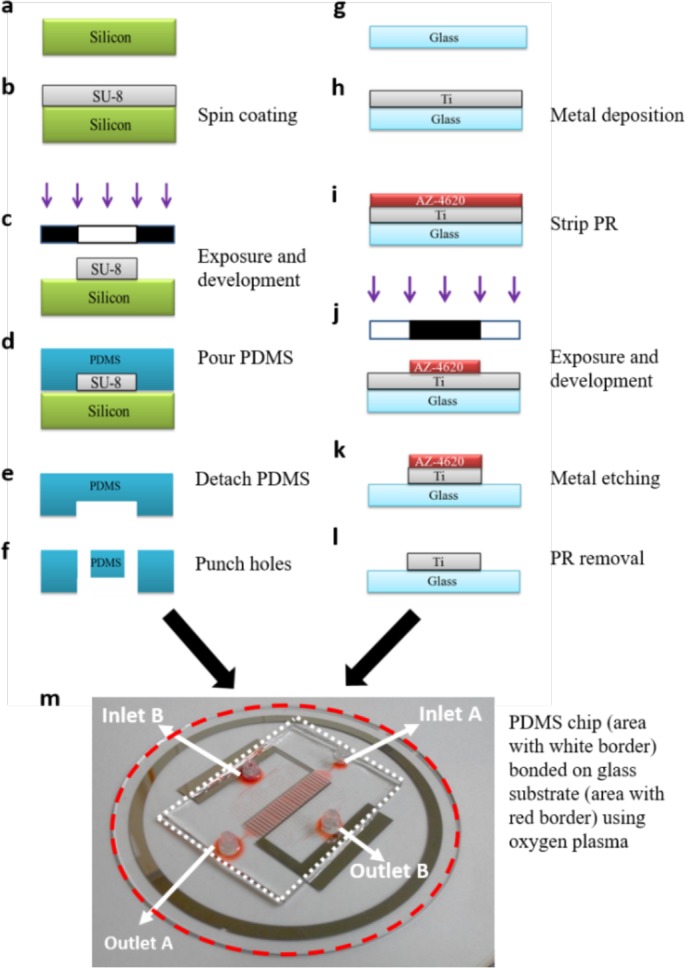
Fabrication of microfluidic chip **a-f.** Fabrication steps for PDMS chip **g-i.** Fabrication steps for metal electrode **m.** Final chip after bonding.

Microfluidic device was developed using soft lithography technique. Master mold for rapid cell trapping microchannel was created using SU8. SU-8 photoresists (SU-8 2015, MicroChem Corp., Newton, Massachusetts) was spun at 2000 rpm for 30 s to get the master mold with 20 μm pillar height. The wafer was then UV-exposed through a glass mask with microfluidic channels with cell traps. UV-exposure was followed by development and baking. SU-8 molds were hard-baked at 150°C for 30 mins (Fig. 5a–5c). Microstructures were cast by using PDMS. The elastomer base and the curing agent (Sylgard 184, Dow Corning Corporation, Midland, Michigan) were mixed in the ratio of 10:1, degassed in vacuum chamber to remove the bubble inside to make the applicable PDMS. The mixed PDMS was then poured onto master mold and heated at 60°C in an oven for two hours. Finally, the holes were punched mechanically through the solid and detached PDMS top cover for the purpose of fluidic connections to outside tubing (Fig. 5d–5f).

A 4 inch glass wafer was piranha cleaned followed by titanium deposition of 2000 Å using E-Gun evaporation for the fabrication of electrodes. The wafer was coated with positive photoresist (Fig. 5g–5i). The wafer was patterned with sawtooth shaped interdigitated electrodes using standard photolithography process and further developed to get desired electrode pattern. Unwanted metal was etched using metal etchant (Fig. 5j–5l). PDMS chip was finally plasma bonded with electrode wafer with proper alignment of microfluidic trapping channels and electrodes. Inlet and outlet connections were made from the punched holes using high quality flexible polymer tubing.

### Cell culture

A549 (ATCC^®^ CCL185™) is a human lung carcinoma cell line and THP-1 (ATCC^®^ TIB202™) is a human peripheral blood acute monocytic leukemia cell line. The cells were maintained in a standard cell culture incubator (5% CO_2_, 95% humidity, 37°C). THP1 cells were cultured in Roswell Park Memorial Institute (RPMI) 1640 medium supplemented with 10% Fetal Bovine Serum (FBS), 4.5 g/L glucose, 10 mM HEPES and 1.0 mM sodium pyruvate, supplemented with 0.05 mM 2-mercaptoethanol.

A549 cells were cultured in 90% Ham's F12K medium supplemented with 10% fetal bovine serum (FBS; Invitrogen). The pH of culture medium containing with 2 mM L-glutamine was adjusted to 7.2 by NaOH and HCl.

We studied the pairing and fusion process with the help of fluorescence labeled cells. A549 cells were labeled with green fluorescence using CMFDA with excitation/emission wavelengths of 492/517 nm and THP-1 cells were labeled with red fluorescence CMTMR with excitation/emission wavelength of 541/565 nm.

### Cell electrofusion experiment

The cell electrofusion chip testing platform consists of a fluorescent microscope (CKX41, Olympus, Tokyo, Japan), a syringe pump (KDS230, KDScientific), used for the sample pumping and sample extraction, a function generator (33220A, Agilent Technology, Santa Clara, California) to apply pairing and fusion signal. Cell pairing and fusion process was recorded as a series of bitmap images by a microscope digital camera (SPOT RT3, Diagnostic Instruments, Sterling Heights, Michigan) connecting to the computer using vendor software SPOT Advanced (ver. 4.6, Diagnostic Instruments, Sterling Heights, Michigan). The microchannels were first washed with 75% ethanol followed by channel cleaning with distilled-deionized (DD) water and removal of air bubbles. In order to reduce the adhesion of cells, 1% Bovine Serum Albumin (BSA) with DD water solution was injected into the chamber to modify the surface properties of microchannel. The fusion buffer was injected to clean the channel again, before loading the cells. The cell with fluorescent marker were well suspended in fusion buffer with concentration of 3 × 10^4^ cells/ml before loading the cells.

**Figure 6:**
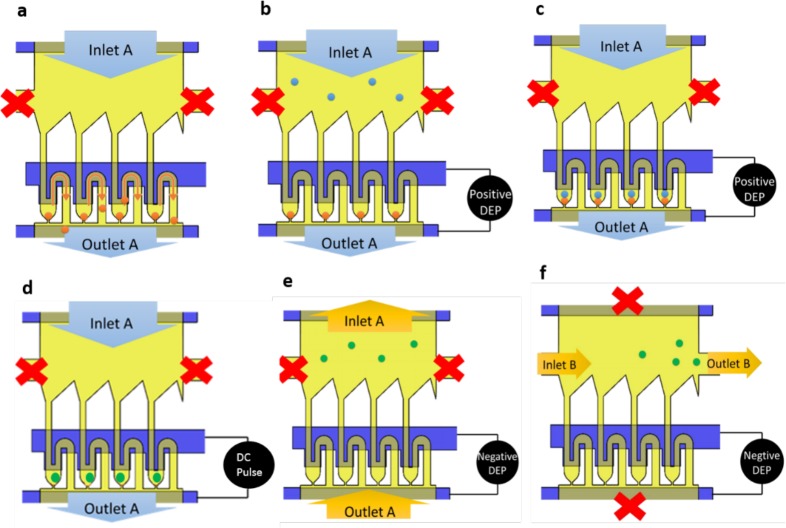
Cell loading and unloading protocol **a.** load type A cell **b.** load type B cells with pDEP activated **c.** cells are paired in trapping channel **d.** apply DC pulse for cell fusion **e.** apply nDEP and insert fusion buffer from outlet B to take out cells **f.** insert fusion buffer from inlet B to collect cells at outlet B.

The process of cell fusion consists of loading of first type of cells i.e. THP1 cells from Inlet A suspened in fusion buffer (Fig. 6a). The syringe pump was turned on and off in regular interval to control the cell population. The extra cells present in the channels were taken out by flowing buffer solution through Inlet B. During the next step, A549 cells were introduced into the micro-channel with flow rate of 0.3 μm/min until it filled an entire channel. The pump was then turned off, and an alignment signal (V_p-p_: 10 V, frequency: 1 MHz) was imposed between the microelectrode arrays at the bottom. The fusion buffer helps to have a low conductivity which is necessary for the dieletrophoresis phenomenon to occur. The low conductivity medium will help to have better cell viability [47]. Driven by the induced positive DEP force, cells inside the microchannel are attracted toward the microelectrodes and are aligned as pairs with a high efficiency (Fig. 6b, 6c). After the cell alignment, DC electric field (amplitude: 2kV/cm, frequency: 10 Hz, duration: 100 μs, and the number of pulses: 10) was applied to induce temporary cell membrane perforation (Fig. 6d). The optimum value of DC field helps in cell membrane reconstruction because of cell's self-recovery ability and resealing of cell membrane after cytoplasm exchange of the paired cells by maintaining cell viability.

The fused cells can be removed by pumping buffer solution through Outlet A and applying negative DEP force (Fig. 6e). Negative DEP helps to move cells away from the pairing channel. Once the fused cells are moved away from the pairing chamber, they can be taken out of the chip by flowing buffer solution from Inlet B and collecting it at Outlet B (Fig. 6f).

## RESULTS AND DISCUSSION

An AC electric field imposed on a freely suspended cell would induce a dipole moment within the cell and result in a potential difference across the plasma membrane similar to capacitive charging building up a potential V_m_ across the membrane. This induced voltage might lead to the membrane permeabilization due to an electrical breakdown on the plasma membrane when the membrane potential difference approximately exceeds 1 V at room temperature [23, 46]. Exposing biological cells to such a high electric-fields environment might lead to a variety of profound biochemical and biophysical effects, such as apoptosis and cell-lysis. One of the challenge for dielectrophoresis is that the normal cell-culture buffer usually has a high electrical conductivity and is unsuitable for DEP manipulation. The normal biological buffer with a high electrical conductivity was accompanied by two drawback effects under normal DEP operation: (i) the heating of the solution; (ii) the electrolytic process (gas bubble formation). Therefore, the low-conductivity medium, which could also decrease the induced transmembrane potential, is favorable for the DEP manipulation of the cells because field-induced apoptosis hardly occurs under low conductivity medium. In addition, since a high-frequency AC potential (above sub-megahertz) could alleviate the induced potential more than a low-frequency one, the manipulation of the cells was operated in a low conductivity medium with an AC potential of 1 MHz to provide a soft electric environment and minimize the stress on the delicate cells. Thus, there are number of factors which are responsible for the process of cell fusion. We studied various parameters and optimized them for the best possible performance in our chip. Effects of these parameters are discussed in further section.

### Cell pairing and fusion

**Figure 7:**
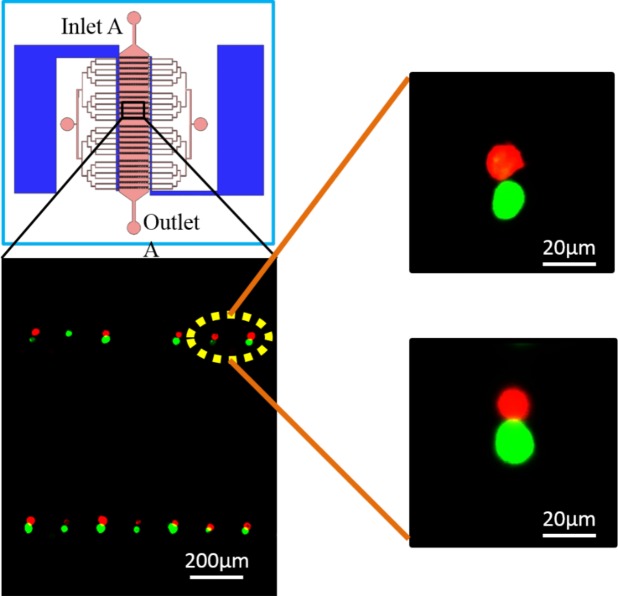
Cell pairing results in fluorescence field A549 cells were labeled with green CMFDA and THP-1 cells were labeled with red CMTMR.

The cell pairing and fusion phenomenon can be observed by imaging and counting the number of cells that exchanged fluorescent molecules. After A549 (marked with green fluorescence marker) cells were trapped by microstructure and THP-1 (marked with red fluorescence marker) cells were aligned by positive DEP force, a series of high electric field pulses were applied to the paired cells accordingly. The process made the cell membrane plasma unstable and formed reversible membrane pores, leading cells in physical contact to achieve electrofusion (Fig. 7)

**Figure 8:**
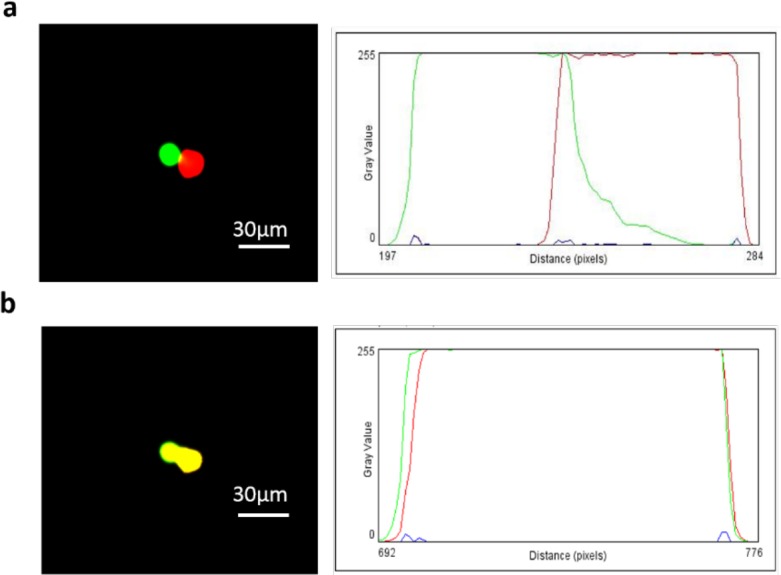
The cell pairing results in fluorescence field **a.** A549 cells were labeled with green CMFDA and THP-1 cells were labeled with red CMTMR **b.** color changes to yellow after complete cell fusion.

The exchange of fluorescence occurred after cell membrane perforation. We confirm complete cell fusion by using ImageJ to analyze the quality of electrofusion process by RGB color model. Green, Red and yellow colors can be represented by their RGB codes as (0, 255, 0), (255, 0, 0), and (255, 255, 0) respectively. The color exchange was observed after application of DC pulse signal. The two kinds of cell in RGB color model were (0, 255, 0) and (255, 0, 0) respectively in the beginning (Fig. 8a). The color changes to yellow (255, 255, 0) after electric field was introduced (Fig. 8b), which indicated complete cell fusion process.

### Effect of flow rate on trapping efficiency

**Figure 9:**
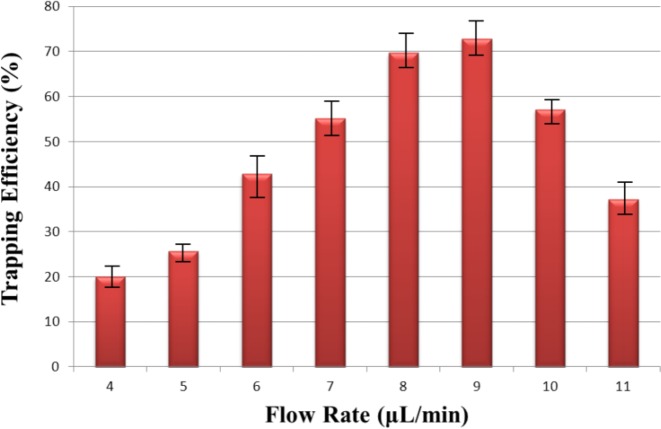
Effect of flow rate on trapping efficiency is calculated The optimum flow rate is observed at 9μl/min at which the maximum trapping efficiency of 73% is observed.

Flow rate is an important factor as cell trapping efficiency relies on the hydrodynamic force. We define the trapping efficiency as the number of successful THP-1 cells trapped using hydrodynamic force, divided by the number of available trapping sites (in this case 960). The larger force would make cells pass through the trapping structure, and the weaker force might cause more than one cell to be trapped in the trapping site. Therefore, the cells were introduced into chip with different flow rate 1–11 μL/min with the increasing interval of 1. The best cell trapping efficiency occurred at the flow rate of 9 μL/min on this chip. (Fig. 9). From the simulations it was observed that in absence of cells, the velocity was about 0.00466 m/s. When the cells are passed in the channels, the velocity was observed to be 0.0024 m/s which represents increase in flow resistance and thus capable of holding the cell in the trap.

### Effect of electric field on pairing efficiency

**Figure 10:**
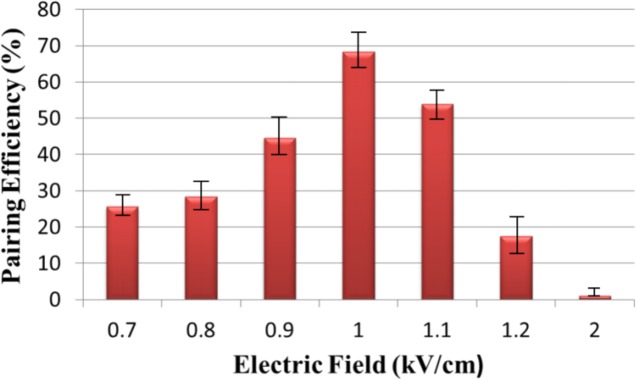
Effect of electric field on pairing efficiency Pairing efficiency of 68% was achieved when electric field was 1kV/cm.

While first type of cells are almost trapped by trapping site, however the DEP signal is necessary to trap other kind of cells into the chip. An AC electric field of high strength and frequency is applied between the two 3D thin film microelectrode arrays. Since the permittivity of the cell cytoplasm, ε_c_, is higher than that of the suspension medium, ε_s_, cells in the microchannel will be driven by the induced positive DEP force to the high electric field region. As from theory, positive DEP is generated at 1 MHz, the frequency of the signal was fixed, which made the strength of electric field become the most effective factor. Electrofusion was carried over a range of values from 0.7 kV/cm to 2 kV/cm. The successful cell pairing is defined when exactly one green A549 cell is paired with one red THP-1 cell. The number of successful pairing is referred as “NP.” The pairing efficiency is defined as (NP/960)*100%, where 960 represents the total trapping sites in one chip. The pairing efficiency of 68% is achieved at electric field around 1 kV/cm (Fig. 10). The percentage of successful cell pairing was more in the middle parts and towards Outlet A but less at the edges.

Meanwhile, the positive DEP force is not strong enough to trap the cell THP-1 when the electric field is below 0.9 kV/cm. However, if the electric field is greater than 1.2 kV/cm, more than one cell was trapped in most trapping sites because the DEP force is very strong. We observed that because of the irregular size of cells and fixed design of hydrodynamic trapping site, there is a loss of cells at the time of trapping of second type of cells.

### Cell electrofusion efficiency

The strength of electric field is one of the significant parameters impacting electrofusion efficiency. When the membrane voltage (V_m_) is over the breakdown voltage, which is around 1 V for most kinds of cells, the cell membrane plasma could not be recovered. While V_m_ was too low, the cell membrane was unable to be perforated. The electric field is varied from 1 kV/cm to 5 kV/cm to see the effect on fusion efficiency. A DC pulse (number of pulses: 10 pulse, duration: 100 μs, frequency: 10 Hz) was used as fusion signal. The fusion efficiency was defined by the number of cells which successfully exchange two kinds of fluorescent (NF) over the number of paired cells (NP), i.e. “electrofusion efficiency = (NF/NP) * 100%”

**Figure 11:**
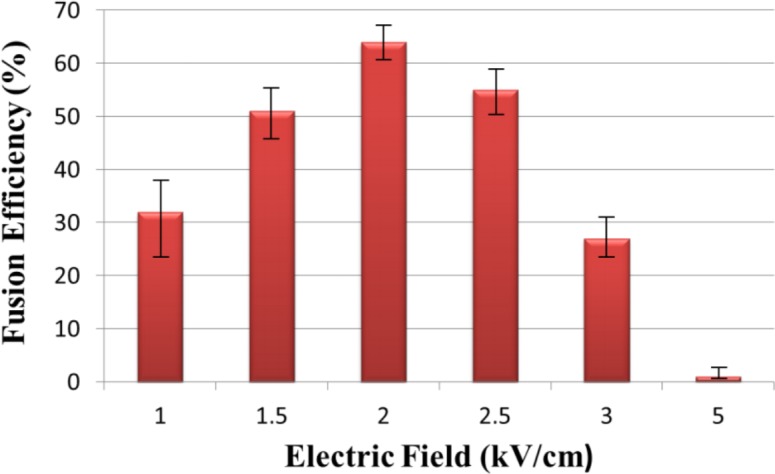
Effect of change in electric field on fusion efficiency The maximum fusion efficiency is obtained at an electric field of 2kV/cm.

Cell electrofusion rate at 1.5 kV/cm was really low, and the highest fusion efficiency was at electric field of 2 kV/cm. Irreversible breakdown of cell membrane was observed when electric field was over 2 kV/cm. However, when the strength of electric field was over 5 kV/cm, most of cells were broken since the electric field induced the cell membrane unable to recover. Therefore, the best electrofusion efficiency on this chip can achieve 64% at electric field around 2 kV/cm (Fig. 11)

### Flow cytometry

Before electrofusion experiments, THP-1 and A549 cells were labelled with CMTMR and CMFDA dyes respectively. After fusion, THP-1/A549 fusions were detected by flow cytometry. The percentage of THP-1/A549 fusion (CMTMR and CMFDA double positive cells) were determined by CellQuest.

**Figure 12:**
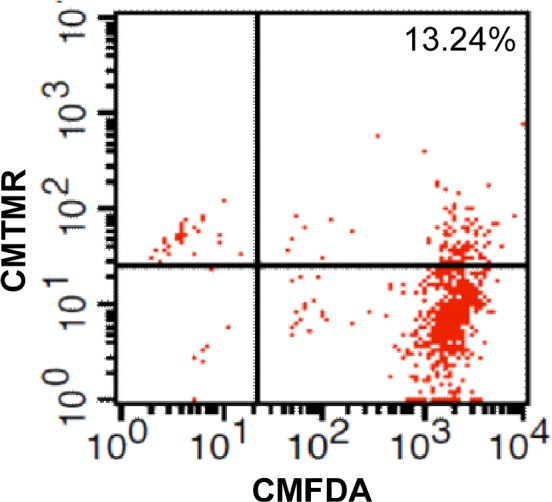
FACS analysis of THP-1/A549 fusions generated by electrofusion THP-1 cells were labeled with CMTMR and were fused with CMFDA-labeled A549 cells.

After electrofusion, all cells were taken out of the chip and were examined by flow cytometry. Electrofusion of THP-1 and A549 cells generated THP-1/A549 fusions that expressed both CMTMR and CMFDA fluorescence (Fig. 12). Since untrapped or unpaired cells were also remained in the chip, the percentages of THP-1/A549 fusions were about 13.24%. The present low percentage mainly results from these untrapped/unpaired cells remained in the chip. Even though these untrapped/unpaired cells were remained in our chip, the yield of fusions cells generated by our chip was similar to that generated by common electrofusion methods (10–20%). The modification of operation process and chip design is undergoing to wash away the unpaired cells before electrofusion process to purify the fused cells for sequent collection.

## CONCLUSIONS

We have designed, simulated, and fabricated a cell electrofusion chip integrated with titanium electrodes on glass wafer. The electrodes are used to generate electric field which offers superior control for locally shaping the field by controlling voltage. The cell electrofusion chip was tested using A549 and THP-1 cell line. The chip can achieve pairing efficiencies of 68% and the fusion rate of 64%. After electrofusion, all cells can be easily taken out than traditional structure array. One limitation of our chip is that some untrapped or unpaired cells remain in the flow channel, which would reduce the yield of fusions cells. To solve this problem, a wash step can be added to eliminate the unpaired cells before electrofusion process. The experimental procedure is simple and efficient and is repeatable. This chip can improve the current cell fusion techniques and overcome the key barriers to be able to build and develop an automated, large and efficient dendritic cell /tumor fusion vaccine therapy
